# Effects of chronic variable stress on cognition and *Bace1* expression among wild-type mice

**DOI:** 10.1038/tp.2016.127

**Published:** 2016-07-12

**Authors:** Z A Cordner, K L K Tamashiro

**Affiliations:** 1 Cellular and Molecular Medicine Graduate Program, Department of Psychiatry and Behavioral Sciences, Johns Hopkins University School of Medicine, Baltimore, MD, USA

## Abstract

Stressful life events, activation of the hypothalamic–pituitary–adrenal (HPA) axis and glucocorticoids are now thought to have a role in the development of several neurodegenerative and psychiatric disorders including Alzheimer’s disease (AD) through mechanisms that may include exacerbation of cognitive impairment, neuronal loss, and beta-amyloid (Aβ) and tau neuropathology. In the current study, we use a wild-type mouse model to demonstrate that chronic variable stress impairs cognitive function and that aged mice are particularly susceptible. We also find that stress exposure is associated with a 1.5- to 2-fold increase in the expression of *Bace1* in the hippocampus of young adult mice and the hippocampus, prefrontal cortex and amygdala of aged mice. Further, the increased expression of *Bace1* was associated with decreased methylation of several CpGs in the *Bace1* promoter region. In a second series of experiments, exposure to environmental enrichment (EE) prevented the stress-related changes in cognition, gene expression and DNA methylation. Together, these findings re-affirm the adverse effects of stress on cognition and further suggest that aged individuals are especially susceptible. In addition, demonstrating that chronic stress results in decreased DNA methylation and increased expression of *Bace1* in the brain may provide a novel link between stress, Aβ pathology and AD. Finally, understanding the mechanisms by which EE prevented the effects of stress on cognition and *Bace1* expression will be an important area of future study that may provide insights into novel approaches to the treatment of AD.

## Introduction

Stressful life events, activation of the hypothalamic–pituitary–adrenal (HPA) axis and glucocorticoids are now thought to have a role in the development of several neurodegenerative and psychiatric disorders including Alzheimer’s disease (AD).^1, 2, 3, 4, 5, 6, 7, 8, 9^ In AD, prior studies using transgenic models indicate that stress exposure might exacerbate beta-amyloid (Aβ) and tau neuropathology,^10, 11^ and it is now thought that two genes whose products are centrally involved in Aβ pathology, *App* and *Bace1*, contain response elements bound by glucocorticoid receptors.^10, 12^ *App* codes for the amyloid precursor protein (APP) and *Bace1* codes for the beta-secretase enzyme, which cleaves APP in the first step of the pathway leading to Aβ peptides and β-amyloid plaques. Further evidence suggesting central roles for stress and HPA axis dysfunction in the development of neurodegenerative disorders comes from animal studies that have successfully prevented or slowed the development of cognitive impairment and neuropathology by means of stress-reducing environmental enrichment (EE) or drugs that block HPA axis activity.^13, 14, 15, 16^ In addition, it is thought that critical developmental periods exist during which individuals are most vulnerable to these detrimental effects of stress,^5, 17, 18, 19, 20, 21^ and old age has been identified as a particularly sensitive time.^1, 4, 5, 7, 18, 19, 20, 21^ Interestingly, prolonged or chronic activation of the HPA axis has been consistently associated with neuronal loss, decreased neurogenesis and altered neural connectivity^5, 7, 18, 19, 20, 22, 23, 24, 25^ and, although underlying mechanisms remain less well understood, stress exposure has also been strongly linked to alterations in epigenetic markers that may ultimately lead to changes in gene expression. As just one example, it is now clear that exposure to glucocorticoids can induce epigenetic modifications of the *Fkbp5* gene, which codes for a protein that normally functions as a co-chaperone of the glucocorticoid receptor,^26, 27^ but, in AD, it may also facilitate tau aggregation.^28, 29, 30^ In addition, epigenetic modifications have been strongly implicated in the regulation of *Bdnf*,^31, 32, 33, 34^ and decreased expression may be involved in a number of neurodegenerative disorders.^35, 36^ Furthermore, several studies have found global changes in epigenetic markers in AD,^37, 38, 39^ a recent genome-wide analysis found DNA methylation (DNAm) differences in post-mortem AD brains at a single locus,^40^ and a study using transgenic mice indicated that chromatin modification might regulate the expression of *Bace1*.^41^

Taken together, prior evidence suggests a role for stress and potential underlying epigenetic mechanisms in the pathogenesis of neurodegenerative disorders including AD. Further, evidence suggests that aging individuals may be particularly sensitive to these adverse effects. Here, young adult and aged wild-type mice were exposed to 14 days of chronic variable stress (CVS) or control conditions. Throughout the study, outbred mice were utilized, rather than an inbred strain or a transgenic model of AD, in order to better reflect the genetic diversity of the human population that is susceptible to cognitive impairment and AD, nearly all cases of which occur sporadically. In this study, we report that CVS results in mild impairments among young adult mice and dramatic impairment among aged mice in two tests of learning and memory. In the hippocampus, we found that CVS is associated with increased expression of *Bace1*, and decreased DNAm of the *Bace1* promoter. Finally, we show that EE prevents stress-related cognitive deficits as well as changes in *Bace1* expression and DNAm. Together, these findings re-affirm the adverse effects of stress on cognition, provide evidence that *Bace1* expression may be epigenetically regulated and sensitive to glucocorticoids, and suggest that understanding mechanisms underlying the effects of EE may eventually lead to novel approaches for the treatment of AD.

## Materials and methods

### Animals

Sixty male CD-1 mice (6 or 18 months old, Charles River, Raleigh, NC, USA) were used and randomly assigned to experimental groups. All mice were individually housed in standard cages (52 × 32 × 24 cm) on a 12 h:12 h light–dark cycle with *ad libitum* access to water and Harlan 2018 chow. All mice were handled daily and housed in the same room except during stress procedures, behavioral testing and sample collection. All protocols were approved by the Animal Care and Use Committee of the Johns Hopkins University School of Medicine.

### Chronic variable stress

CVS is a commonly used paradigm designed to introduce recurrent physical, psychological and social stress that is unpredictable and unavoidable.^42, 43, 44, 45, 46^ In the CVS paradigm used here, mice were exposed to one stressor each day for 14 days (Supplementary Table S1). In the first experiment, mice were maintained under control conditions (CTRL) or exposed to CVS (Stress) at either 6 months (Young) or 18 months of age (Aged), which resulted in four groups: Young CTRL (*n*=6), Young Stress (*n*=6), Aged CTRL (*n*=8) and Aged Stress (*n*=8).

### Environmental enrichment

In a separate cohort, mice were exposed to control conditions, CVS, or CVS and EE. EE included a large tub cage (104 × 56 × 48 cm) with extra bedding, nesting sheets, polycarbonate tunnels, balls and housing domes (Bio-Serv, Frenchtown, NJ, USA). EE was started 1 week before CVS and continued throughout the 14 days of stress. This resulted in the following six groups: Young CTRL (*n*=5), Young Stress (*n*=5), Young Stress+EE (*n*=6), Aged CTRL (*n*=5), Aged Stress (*n*=5) and Aged Stress+EE (*n*=6).

### Plasma CORT response to acute restraint stress

At the onset of the light period, all mice were moved from the housing facility to a separate testing room and allowed to habituate for 1 h. After the habituation period, baseline tail blood samples were collected from all mice on days 1 and 14. Stressed mice were then exposed to 30 min of restraint (unstressed mice were returned to their home cages) after which a second sample was collected. A third sample was collected 1 h later. Plasma CORT concentration was determined by radioimmunoassay (MP Biomedicals, Solon, OH, USA), and area under the curve was calculated.

### Behavioral testing

Following 14 days of CVS or control conditions, behavior was assessed in the open field (OF), novel object recognition (NOR) test and Barnes maze. All mice were first tested in the OF. The following 2 days served as habituation for the NOR test. One day after habituation, mice were tested in the ‘acquisition’ trial of the NOR. Twenty-four hours after acquisition, mice were tested in the ‘recall’ trial of the NOR. Mice were then left undisturbed for 2 days, followed by five consecutive days of testing in the Barnes maze. Within each behavioral test, the order in which mice were tested was randomly determined.

### Open field

The OF consists of a 60-cm square plastic chamber with a clearly marked inner zone. Each mouse was allowed to explore the OF for 10 min. Behavior was coded by a blinded observer for time spent in the inner zone and time spent exploring, immobile, assessing risk and grooming.

### NOR test

Mice were habituated to the testing chamber for 10 min a day for 3 days. On day 4, two objects were placed in the chamber and each mouse was allowed to explore for 10 min (‘acquisition’ trial). On day 5, one ‘familiar’ object was replaced with a ‘novel’ object and each mouse was again allowed to explore for 10 min (‘recall’ trial). The location of the novel object was counterbalanced across all groups. Behavior was coded by a blinded observer. During the acquisition trial, time spent exploring either object was recorded. During the recall trial, times spent exploring the novel and familiar objects were recorded. Exploration of an object was defined as being within 2 cm of contact while facing an object.

### Barnes maze

The Barnes maze consists of a plastic circular, elevated platform (122 cm diameter) with 40 holes (5 cm diameter) around its periphery. A hidden escape box is fixed under one hole. Three visual cues and a bright light are fixed around the perimeter. Mice were allowed to explore the maze during four trials a day for four consecutive days with 30 min between trials. On the fifth day, each mouse was given a single trial. If a mouse failed to find the escape box within 180 s, it was gently guided to the escape. Latency to entering the escape box was measured for each trial. As a measure of exploratory behavior on the Barnes maze, the number of errors per minute was measured during the first trial. An error was defined as a nose poke into a hole that did not contain the escape box.

### Tissue collection

After behavioral testing, all mice were killed by rapid decapitation. The adrenal glands were weighed. Brains were removed, immediately frozen on powdered dry ice and stored at −80 °C. The hippocampus, prefrontal cortex (PFC) and amygdala were isolated from 300-μM-thick frozen coronal sections using a blunted 16-gauge needle according to previously described methods.^26^ For each mouse, tissue from the right side of the brain was used for gene expression analysis and tissue from the left side was used for DNAm analysis.

### Gene expression

Tissue punches were placed in Qiazol. Total RNA was extracted using the RNeasy Lipid Tissue Mini Kit (Qiagen, Valencia, CA, USA). Complementary DNA was generated using the QuantiTect Reverse Transcription Kit (Qiagen). Quantitative real-time PCR was carried out in triplicate using TaqMan Master Mix and probes (Applied Biosystems, Foster City, CA, USA). Expression relative to *Actb* was determined by the −ΔΔCt method. TaqMan probes for genes of interest are listed in Supplementary Table S2.

### Bisulfite pyrosequencing

Genomic DNA was isolated using the Masterpure DNA Purification Kit (Epicentre, Madison, WI, USA). Bisulfite conversion was carried out using the EZ-DNA Methylation-Gold Kit (Zymo Research, Irvine, CA, USA). Bisulfite-treated genomic DNA was then used for nested PCR. Nested PCR products were mixed with streptavidin-coated sepharose beads (GE Healthcare, Waukesha, WI, USA), isolated with a vacuum prep workstation and released into PSQ HS 96-well plates containing pyrosequencing primers. PyroMark Gold Reagents and a PyroMark MD System (Qiagen) were used for pyrosequencing. Quantification of methylation was performed with Pyro Q-CpG v1.0.9 (Qiagen). Sequences of primers are listed in Supplementary Table S3.

### Statistical analysis

Statistical analysis was completed using Statistica 7 (StatSoft, Tulsa, OK, USA). Data are expressed as averages±s.e.m. In the first cohort, differences between groups were assessed by factorial or repeated measures analysis of variance with ‘Age’ and ‘Stress’ as between subject factors followed by Tukey's *post hoc* analysis. In the second cohort, differences between groups were assessed by main effects or repeated measures analysis of variance, followed by Tukey's *post hoc* analysis. Correlations were assessed by Pearson’s correlation. For all tests, *P*<0.05 was considered significant. The number of animals needed per group was estimated by power calculation to detect statistically significant differences (*P*<0.05) at ⩾80% power.

## Results

### CVS results in cognitive impairment, and aged mice are especially susceptible

**Figure 1:**
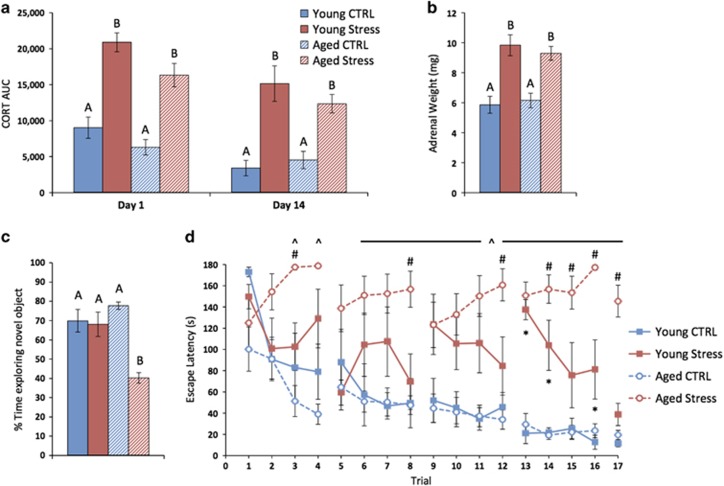
Chronic variable stress (CVS) impairs performance in tests of cognitive function; aged mice are particularly susceptible. (**a**) Restraint stress tests on days 1 and 14 resulted in elevation of plasma corticosterone (CORT) among young adult and aged mice. (**b**) Chronic overproduction of stress hormones in response to CVS is reflected by adrenal hypertrophy among stressed mice. (**c**) Behavioral tests began after 14-day CVS. CVS resulted in impaired performance in the novel object recognition (NOR) test only among aged mice. (**d**) In the Barnes maze, CVS resulted in increased escape latency among young adult and aged mice, but aged mice were more profoundly impaired. Data represent mean±s.e.m. For *post hoc* analysis in **a**–**c**, groups that do not share letters are significantly different (*P*<0.05). For **d**, *P<0.05 Young Stress versus CTRL; ^*P*<0.05 Aged Stress versus CTRL; #*P*<0.05 Young Stress versus Aged Stress.

On days 1 and 14, plasma CORT levels were determined at baseline, immediately following 30 min of restraint (control mice remained in their home cages), and after a 60-min recovery period. There were no effects of age or stress on baseline or recovery CORT levels (Supplementary Figure S1a, b). However, area under the curve analysis shows that restraint resulted in elevation of plasma CORT among stressed mice on day 1 (*P*<0.0001) and day 14 of CVS (*P*<0.0001). Repeated measures analysis of variance revealed that CORT responses were slightly lower on day 14 (*P*<0.0001; Figure 1a). The chronic overproduction of stress hormones in response to CVS was further reflected by adrenal hypertrophy among all stressed mice at the end of the experiment (*P*<0.0001; Figure 1b).

After CVS, behavior was assessed in the OF, NOR test and Barnes maze. There was no effect of age or stress on behavior in the OF (Supplementary Figure S2a). In the acquisition trial of the NOR test, there were no effects of age or stress on time spent exploring (Supplementary Figure S2b). In the memory recall trial, Young CTRL and Aged CTRL mice were indistinguishable while CVS resulted in decreased NOR only among aged mice (*P*=0.002; Figure 1c). In the first trial of the Barnes maze, there were no effects of age or stress on exploratory behavior (Supplementary Figure S2c). Across subsequent trials, Young CTRL and Aged CTRL mice were again indistinguishable while CVS resulted in increased escape latency among both young adult and aged mice (*P*<0.0001). However, Aged Stress mice required significantly more time to complete the maze compared with all other groups (*P*=0.002). *Post hoc* analysis indicated that Young Stress mice had increased escape latency compared with both control groups during 3 of 17 trials, whereas significant differences between the Aged Stress group and control groups were found during 14 of 17 trials (*P*<0.05). Moreover, Aged Stress mice required significantly more time to find the escape than Young Stress mice during 7 of 17 trials (*P*<0.05; Figure 1d).

### CVS increases *Bace1* expression

**Figure 2:**
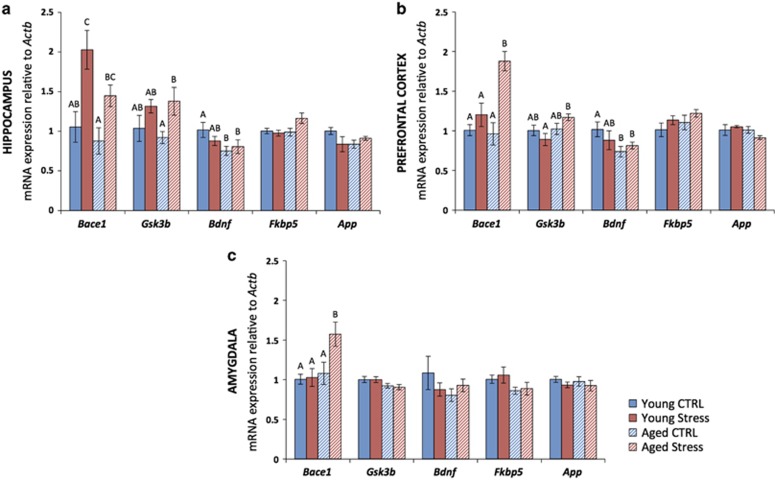
Chronic variable stress (CVS) results in increased expression of *Bace1*. (**a**) In the hippocampus, CVS was associated with increased expression of *Bace1* among both aged and young adult mice. CVS was also associated with increased expression of *Gsk3b*, and the expression of *Bdnf* was significantly lower among aged mice. There was a trend toward increased expression of *Fkbp5* among aged mice exposed to CVS. (**b**) In the prefrontal cortex (PFC), CVS was associated with increased expression of *Bace1* only among aged mice. There was also an effect of age on the expression of *Gsk3b* and the expression of *Bdnf* was significantly lower among aged mice. (**c**) In the amygdala, the expression of *Bace1* was increased only in aged mice exposed to CVS. Data represent mean±s.e.m. For *post hoc* analysis, groups that do not share letters are significantly different (*P*<0.05).

To determine the effects of age and CVS on stress-related and AD-related gene expression, mRNA levels of *Bace1*, *Gsk3b*, *Bdnf*, *Fkbp5* and *App* were measured in the hippocampus, PFC and amygdala. In the hippocampus, CVS was associated with increased expression of *Bace1* among both aged and young adult mice (*P*=0.006). CVS was also associated with increased expression of *Gsk3b* (*P*=0.02), and *Bdnf* expression was lower among aged mice (*P*=0.04; Figure 2a).

In the PFC, CVS was associated with increased expression of *Bace1* only among aged mice (*P*=0.04). There was also an effect of age on the expression of *Gsk3b* (*P*=0.03). As in the hippocampus, the expression of *Bdnf* was lower among aged mice (*P*=0.04; Figure 2b).

In the amygdala, the expression of *Bace1* was increased only in aged mice exposed to CVS (*P*=0.03; Figure 2c).

### The stress-related increase in *Bace1* expression may be epigenetically regulated

**Figure 3:**
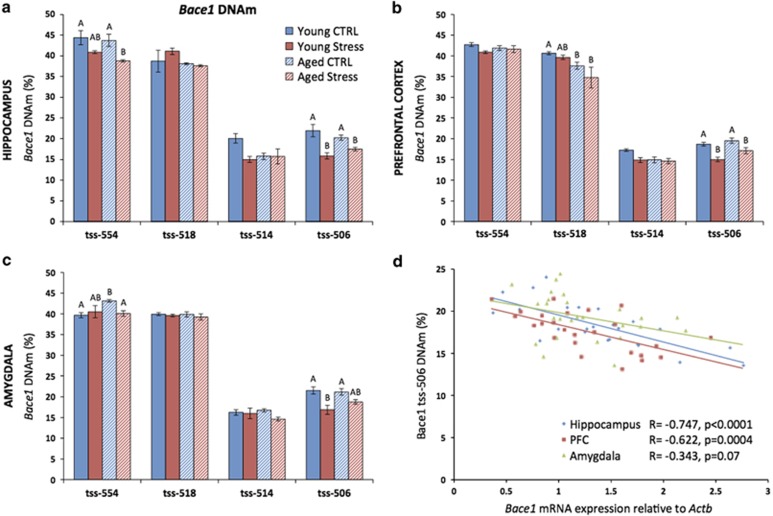
The stress-related increase in *Bace1* expression may be epigenetically mediated. (**a**) In the hippocampus, chronic variable stress (CVS) was associated with demethylation of CpGs located at tss-554 and tss-506. There was a trend toward higher methylation in the Young Control group at tss-514. (**b**) In the prefrontal cortex (PFC), CVS was associated with demethylation of tss-506. Aged mice also had lower methylation at tss-518. (**c**) In the amygdala, CVS was associated with demethylation of tss-506 and Aged Control mice had higher methylation at tss-554. (**d**) The expression of *Bace1* is inversely correlated with DNAm at tss-506 in the hippocampus and in the PFC. The correlation did not reach significance in the amygdala. Data represent mean±s.e.m. For *post hoc* analysis, groups that do not share letters are significantly different (*P*<0.05).

In order to determine whether the observed changes in gene expression in response to CVS might be epigenetically mediated, bisulfite pyrosequencing was performed using primers designed to target CpGs in the promoter regions of *Bace1* and *Gsk3b*, as well as in the region immediately upstream of *Bdnf* exon 4 where DNAm changes have been previously reported.^31^ In the hippocampus, PFC and amygdala, there was a consistent pattern of stress-related decreases in methylation of several CpGs in the promoter region of *Bace1*. Specifically, in the hippocampus, CVS was associated with demethylation of CpGs located at 554 bases upstream of the transcription start site (tss-554; *P*=0.0008) and tss-506 (*P*<0.0001; Figure 3a). In the PFC, CVS was associated with demethylation of tss-506 (*P*=0.0002) and methylation of tss-518 was lower among aged mice (*P*=0.04; Figure 3b). In the amygdala, CVS was again associated with demethylation of tss-506 (*P*=0.0003), and Aged CTRL mice had higher methylation at tss-554 (*P*=0.03; Figure 3c).

Regarding *Gsk3b*, there were no effects of age or stress on the methylation of any CpGs assessed (Supplementary Figure S3). For the *Bdnf* exon 4 region, aged mice had higher methylation at tss-109 in the hippocampus (*P*=0.004). Otherwise, there were no effects of age or stress on methylation of other CpGs (Supplementary Figure S4).

Because gene expression and DNAm were assessed within the same subjects, we were able to correlate the two measures. We found significant inverse correlations between the expression of *Bace1* and methylation at the tss-506 CpG in the hippocampus (*r*=−0.747, *P*<0.0001) and PFC (*r*=−0.622, *P*=0.0004) and a similar, but nonsignificant trend in the amygdala (*r*=−0.343, *P*=0.07; Figure 3d). Interestingly, the tss-506 CpG is located within a putative Sp1 transcription factor-binding site. Sp1 has been previously shown to regulate the expression of human *BACE1* (ref. 47) and binding of Sp1 to its target sequence is thought to be regulated by DNAm.^48^ Further, while not overlapping a CpG, a putative glucocorticoid response element is located nearby at tss-802.

### EE prevents the effects of CVS on cognition and *Bace1* expression

**Figure 4:**
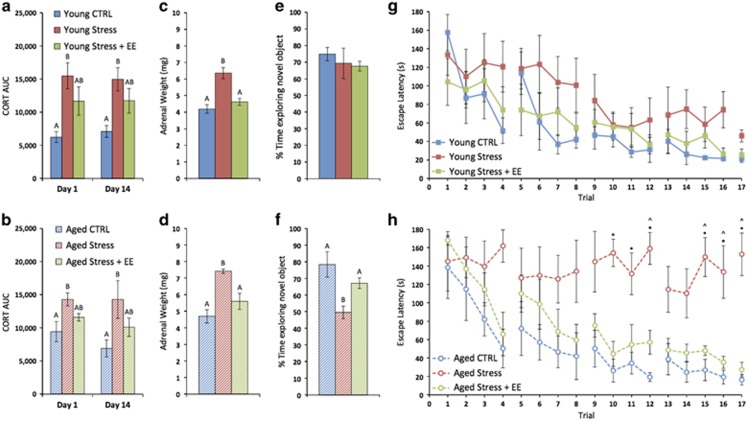
Environmental enrichment (EE) prevents the effects of chronic variable stress (CVS) on cognitive performance among aged mice. (**a**, **b**) Restrain stress tests on days 1 and 14 resulted in elevation of plasma corticosterone (CORT) among young adult and aged mice, whereas EE appeared to blunt the stress response. (**c**, **d**) Chronic overproduction of stress hormones in response to CVS is reflected by adrenal hypertrophy among stressed mice. This effect was prevented by EE. (**e**, **f**) CVS resulted in impaired performance in the novel object recognition (NOR) test only among aged mice. This effect was prevented by EE. (**g, h**) In the Barnes maze, there was a trend for increased escape latency in the Young Stress group. Aged Stress mice had significantly greater escape latency compared with both Aged CTRL and Aged Stress+EE mice. Aged mice exposed to CVS+EE were indistinguishable from controls. For *post hoc* analysis in **a**–**f**, groups that do not share letters are significantly different (*P*<0.05). For **h**, *P<0.05 Aged Stress versus Aged CTRL; ^*P*<0.05 Aged Stress versus Aged Stress+EE.

Because EE has been previously shown to moderate the stress response and enhance cognitive performance in rodent models,^15, 16, 49^ we sought to understand whether EE might prevent the observed effects of CVS on behavior, gene expression and DNAm. In a separate cohort, young adult and aged mice were exposed to control conditions, CVS or CVS+EE. In restraint stress tests on days 1 and 14, as in the first cohort, there were no group effects on baseline or recovery CORT levels (Supplementary Figure S5a–d). Area under the curve analysis revealed that all stressed mice demonstrated elevation of CORT in response to restraint (*P*<0.05); however, there was a trend toward a blunted CORT response among mice exposed to CVS+EE (Figures 4a and b). The potentially protective effects of EE were further supported by measurement of adrenal weight at the end of the experiment. Among young adult mice, there was significant adrenal hypertrophy among Young Stress mice compared with both the Young CTRL group (*P*=0.0006) and Young Stress+EE group (*P*=0.003; Figure 4c). Similarly, there was significant adrenal hypertrophy among Aged Stress mice compared with both the Aged CTRL group (*P*=0.002) and Aged Stress+EE group (*P*=0.004; Figure 4d).

We next assessed the effects of EE on behavior. In the OF, there were no effects among young adult mice and Aged CTRL mice spent slightly longer exploring than Aged Stress mice (*P*=0.04) and Aged Stress+EE mice (*P*=0.01; Supplementary Figure S6a,b). In the acquisition trial of the NOR test, there were again no group effects on time spent exploring (Supplementary Figure S6c,d). In the memory recall trial, as in the first cohort, there was no group effect on the behavior of young adult mice (Figure 4e). However, Aged Stress mice were significantly impaired compared with both Aged CTRL (*P*=0.007) and Aged Stress+EE mice (*P*=0.05; Figure 4f). In the first trial of the Barnes maze, there were again no group effects on exploratory behavior (Supplementary Figure S6e,f). Across subsequent trials, there was an overall effect of time on the performance of young adult mice (*P*<0.0001) and a trend for an overall group effect (*P*=0.08; Figure 4g). Among aged mice, there was a significant time by group interaction (*P*=0.001). *Post hoc* analysis revealed significantly greater escape latency among Aged Stress mice compared with Aged CTRL mice during 6 of the 17 trials (*P*<0.05). Aged Stress mice also had significantly greater escape latency compared with Aged Stress+EE mice during 4 of the 17 trials (*P*<0.05). There were no trials during which the performance of Aged CTRL mice differed significantly from Aged Stress+EE mice, further suggesting that EE may protect against the effects of stress on cognition (Figure 4h).

**Figure 5:**
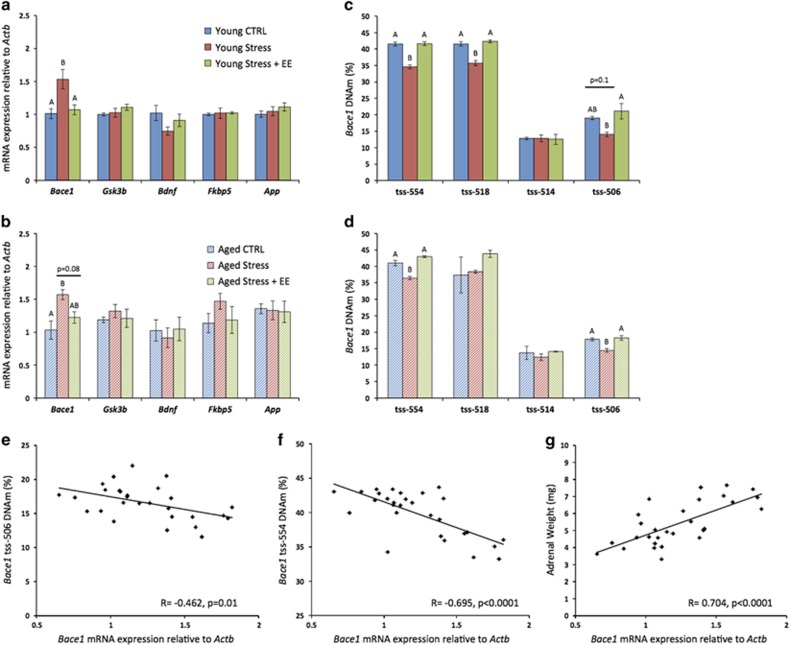
Environmental enrichment (EE) prevents the effects of chronic variable stress (CVS) on *Bace1* expression and DNA methylation (DNAm). (**a**, **b**) In the hippocampus, CVS was associated with increased *Bace1* expression among young adult and aged mice. This effect was prevented by EE. (**c**, **d**) In the hippocampus, CVS was associated with demethylation of *Bace1* promoter region CpGs located at tss-554, tss-518 and tss-506 among young adult mice, and tss-554 and tss-506 among aged mice. All of these effects were prevented by EE. (**e**, **f**) Across all groups, the hippocampal expression of *Bace1* is inversely correlated with DNAm at tss-506 and tss-554. (**g**) The hippocampal expression of *Bace1* is also positively correlated with adrenal weight. Data represent mean±s.e.m. For *post hoc* analysis, groups that do not share letters are significantly different (*P*<0.05).

After behavioral testing, we assessed gene expression and DNAm. Because the most consistent changes in the first cohort were found in the hippocampus, we focused on this brain region in the second cohort. We found that hippocampal *Bace1* expression was higher among Young Stress mice compared with both Young CTRL (*P*=0.01) and Young Stress+EE mice (*P*=0.02; Figure 5a). Similarly, *Bace1* expression was higher among Aged Stress mice compared with Aged CTRL (*P*=0.02), and there was a trend compared with the Aged Stress+EE group (*P*=0.08; Figure 5b). Unlike the first cohort, there was no effect of stress or EE on the expression of *Gsk3b.*

When we assessed DNAm of the *Bace1* promoter region, we again found that CVS was associated with demethylation of several CpGs, and this pattern of demethylation was prevented by EE. Specifically, as in the first cohort, Young Stress mice had lower DNAm at tss-554 and tss-506 compared with both Young CTRL and Young Stress+EE mice (*P*<0.05). In the second cohort, but not the first, Young Stress mice also had lower DNAm at tss-518 compared with both Young CTRL and Young Stress+EE mice (*P*<0.05; Figure 5c). Aged Stress mice had lower methylation at tss-554 and tss-506 compared with both Aged CTRL and Aged Stress+EE mice (*P*<0.05), which is consistent with findings from the first cohort (Figure 5d).

As in the first cohort, we found an inverse correlation between *Bace1* expression and methylation at tss-506 (*r*=−0.462, *P*=0.01; Figure 5e). In the second cohort, there was also a strong inverse correlation between the expression of *Bace1* and methylation at tss-554 (*r*=−0.695, *P*<0.0001; Figure 5f), suggesting again that *Bace1* may be epigenetically regulated and that changes in DNAm may underlie the stress-related increase in *Bace1* expression. Finally, we found that hippocampal expression of *Bace1* was strongly correlated with adrenal weight (*r*=−0.704, *P*<0.0001; Figure 5g), suggesting a possible role for CORT in the regulation of *Bace1*; however, additional studies are warranted to determine the relative contribution of the HPA axis and CORT versus the many other signaling pathways that are concurrently affected by stress exposure. Together, these data largely confirm the findings of the first cohort and further suggest that EE is effective in preventing the effects of CVS on cognition as well as the expression and DNAm of *Bace1*.

## Discussion

The data presented here suggest that stress can lead to cognitive impairment among both young adult and aged mice; however, aged mice appear to be especially sensitive. Specifically, under control conditions, we found no differences in the performance of young adult and aged control mice in two behavioral tests of learning and memory. However, clear differences emerged among mice exposed to 2 weeks of CVS. In young adult mice, stress induced moderate impairment in the Barnes maze, a largely hippocampal-dependent task (reviewed in Cordner and Tamashiro^50^). In aged mice, stress resulted in profound impairment in both the Barnes maze and NOR test, which likely involves both hippocampal and cortical function.^50^ Several other studies have implicated stress in the pathogenesis of cognitive decline and AD, and prior work also suggests that the aging brain may be particularly susceptible (reviewed in refs 1, 4, 51, 52,53). For example, in one study, exposure of young adult mice to chronically high levels of CORT resulted in impaired performance in the NOR test, Barnes maze and Morris water maze.^54^ Other studies have used chronic mild stressors, similar to the CVS procedure used here, and found stress-related impairment in the Morris water maze^55^ and NOR^56^ among young adult mice. Regarding age-dependent effects of stress, one study found that prolonged social stress or administration of CORT impairs Morris water maze performance in middle-aged but not young adult rats,^57^ and another study found that even unstressed levels of CORT might negatively affect cognition in aged rats.^5^

Regarding the effects of stress in the brain, here we show that CVS increased the expression of *Bace1* by 1.5- to 2-fold in the hippocampus of young adult mice and in the hippocampus, PFC and amygdala of aged mice. *Bace1*, which codes for the beta-secretase enzyme, is critically important in AD as it cleaves APP in the first step of the pathway ultimately leading to Aβ peptides and β-amyloid plaques.^58, 59, 60^ Several studies have used transgenic mouse models of AD to demonstrate that chronic stress increases β-amyloid plaque burden^61, 62, 63^ and that delivery of exogenous glucocorticoids can increase the expression of both beta-secretase and APP.^10^ One study using wild-type rats found increased beta-secretase protein expression in response to chronic stress.^64^ However, these prior reports provided limited insight into mechanisms underlying the increase in beta-secretase expression.

Here, we provide novel insight into the potential epigenetic regulation of *Bace1* and suggest that DNAm changes may drive the stress-related increase in *Bace1* expression. Whereas at least one other study has reported that histone modifications regulate *Bace1* expression,^41^ DNAm of *Bace1* has not been well studied. In addition to finding stress-related decreases in methylation of several CpGs in two separate cohorts, we found that *Bace1* expression across all groups strongly correlated with DNAm of a CpG at tss-506. Although this study focused on a small number of CpGs in the *Bace1* promoter region and there are many others that could have regulatory functions, we know from studies of other genes that, in some cases, methylation changes at single CpGs can greatly affect gene expression.^65, 66, 67, 68^ The possibility that the observed changes in DNAm are mediating *Bace1* expression is further supported by the fact that the tss-506 CpG is located within a putative Sp1-binding site, and the presence of a nearby putative glucocorticoid response element suggests that glucocorticoid receptor signaling may also be involved.

Finally, we show that EE prevents the effects of CVS on cognition, *Bace1* expression and promoter methylation. EE has been previously shown to improve cognitive performance of wild-type and transgenic AD models,^16^ reduce Aβ levels in a transgenic AD mouse model,^15^ enhance neuronal plasticity (reviewed in Van Praag *et al.*^69^ and Nithianantharajah and Hannan^70^) and mitigate the effects of stress on cognition in wild-type rats.^49^ Further, numerous clinical reports suggest that enriching/stimulating environments and cognitive exercise may protect against cognitive decline (reviewed in Valenzuela and Sachdev^71^ and Bavelier *et al.*^72^). However, the effects of EE on stress-related cognitive impairment and *Bace1* expression have not been well studied. Finally, although the precise mechanism linking CVS and EE to changes in methylation of the *Bace1* promoter remains an area of ongoing investigation, we also found that *Bace1* expression strongly correlated with adrenal weight, suggesting a role for glucocorticoids, which have been previously associated with changes in DNAm.^73, 74^

Together, the current findings confirm the adverse effects of stress on cognition and further suggest that aged mice are especially susceptible. In addition, we report that CVS decreases methylation and increases expression of *Bace1* in the brain, which may provide a novel link between stress, Aβ pathology and AD. It is important to note that, in the current study, we assessed the effects of CVS on outbred, wild-type mice, which we believe better reflects the genetic diversity of individuals at risk for stress-related cognitive impairment and AD. However, because wild-type mice do not develop typical β-amyloid plaques or neurofibrillary tangles, we are unable to correlate stress exposure or behavior with AD-like pathology in the current study. Moving forward, exploring the mechanisms that link stress exposure to cognitive impairment and changes in methylation and expression of AD-related genes clearly warrant further investigation. Doing so may serve to re-affirm the role of the HPA axis in the β-amyloid pathway and emphasize the need to carefully monitor cognitive function among patients (especially elderly patients) who have been exposed to high levels of stress or treated with glucocorticoids. Finally, understanding the mechanisms by which EE effectively prevented the effects of stress on cognition and *Bace1* expression will be an important area of future study that may ultimately provide insights into novel therapeutic approaches for the treatment of AD.
