# Supplementary figures and images for: Effects of chronic variable stress on cognition and Bace1 expression among wild-type mice
Source: Transl Psychiatry. 2016 Jul 12;6(7):e854–. doi: 10.1038/tp.2016.127 (PMC5545713; doi:10.1038/tp.2016.127)

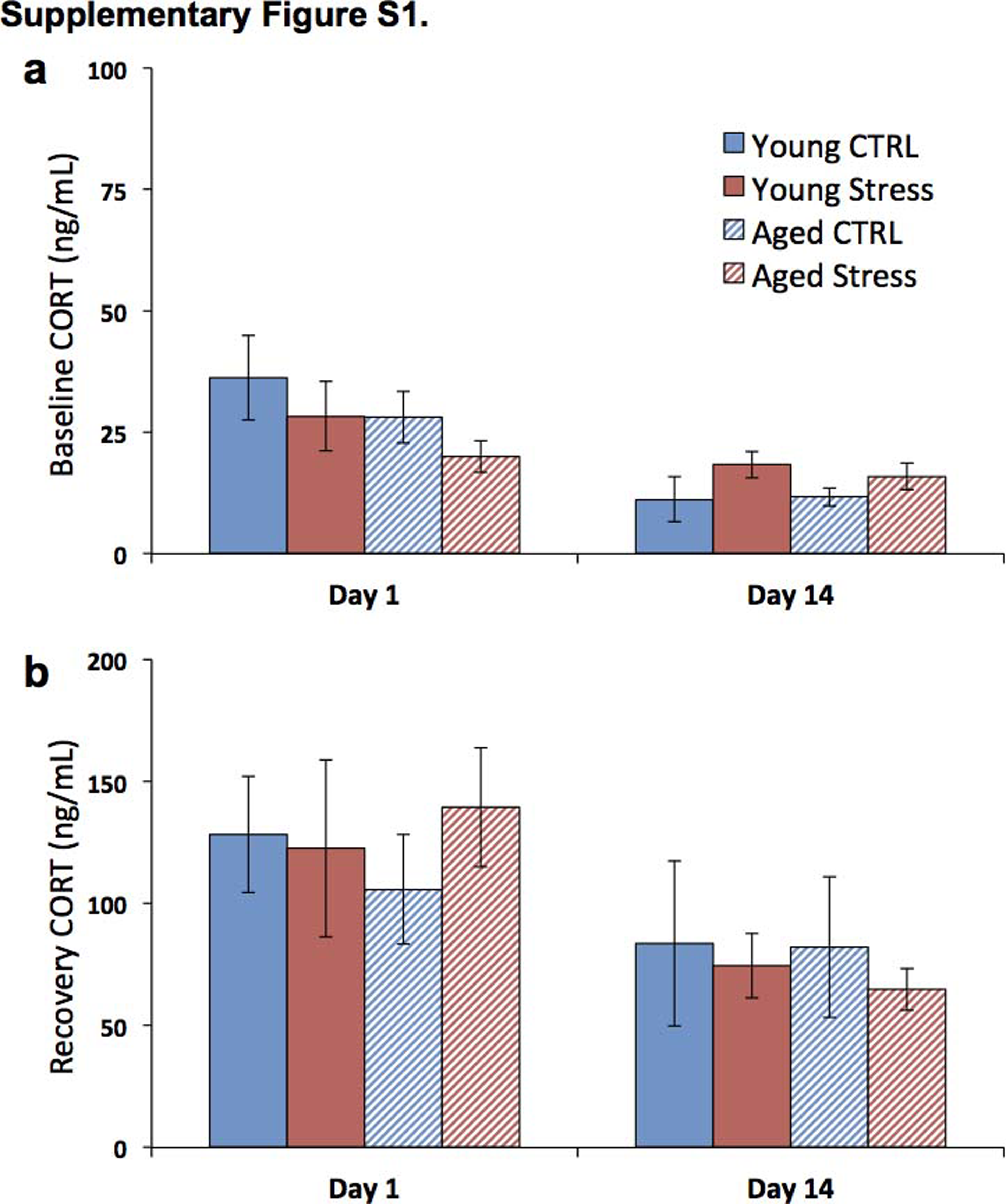

Supplement: Supplementary Figure 1 [file tp2016127x1.tif]

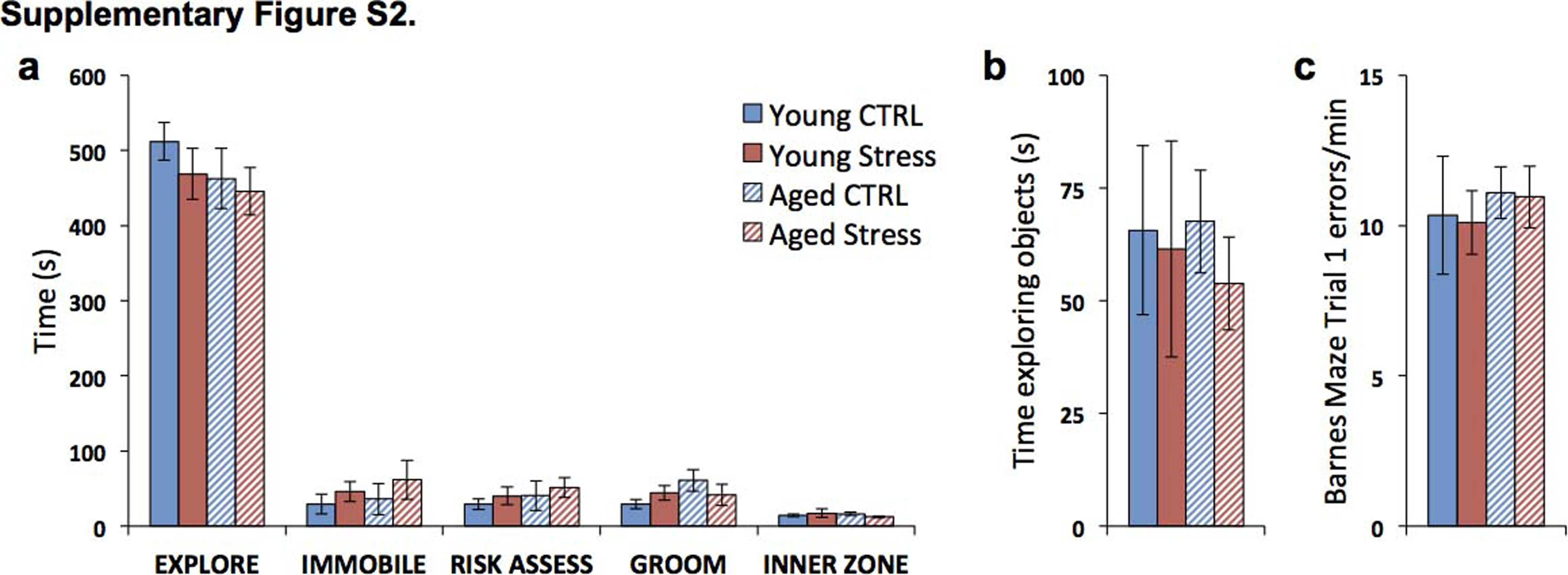

Supplement: Supplementary Figure 2 [file tp2016127x2.tif]

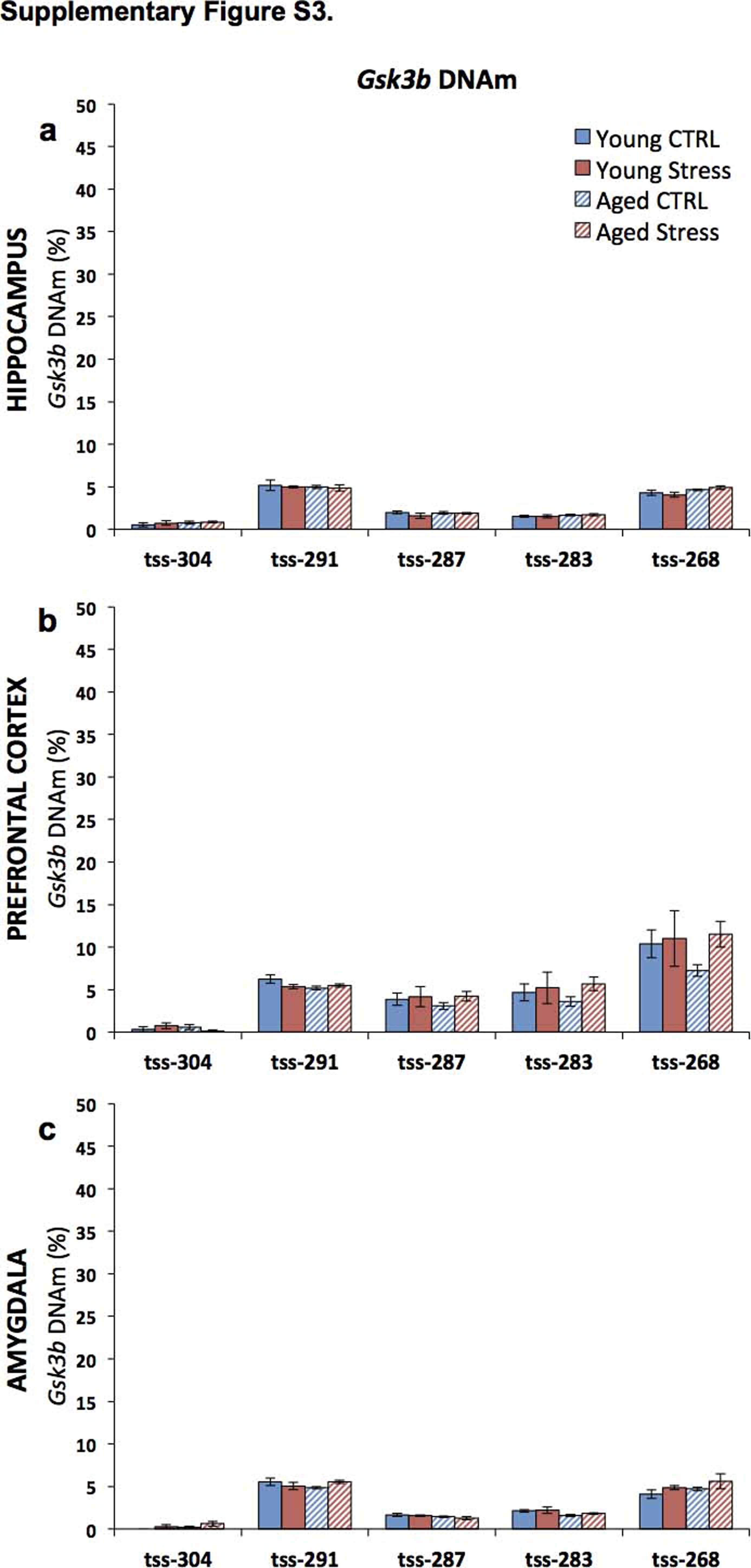

Supplement: Supplementary Figure 3 [file tp2016127x3.tif]

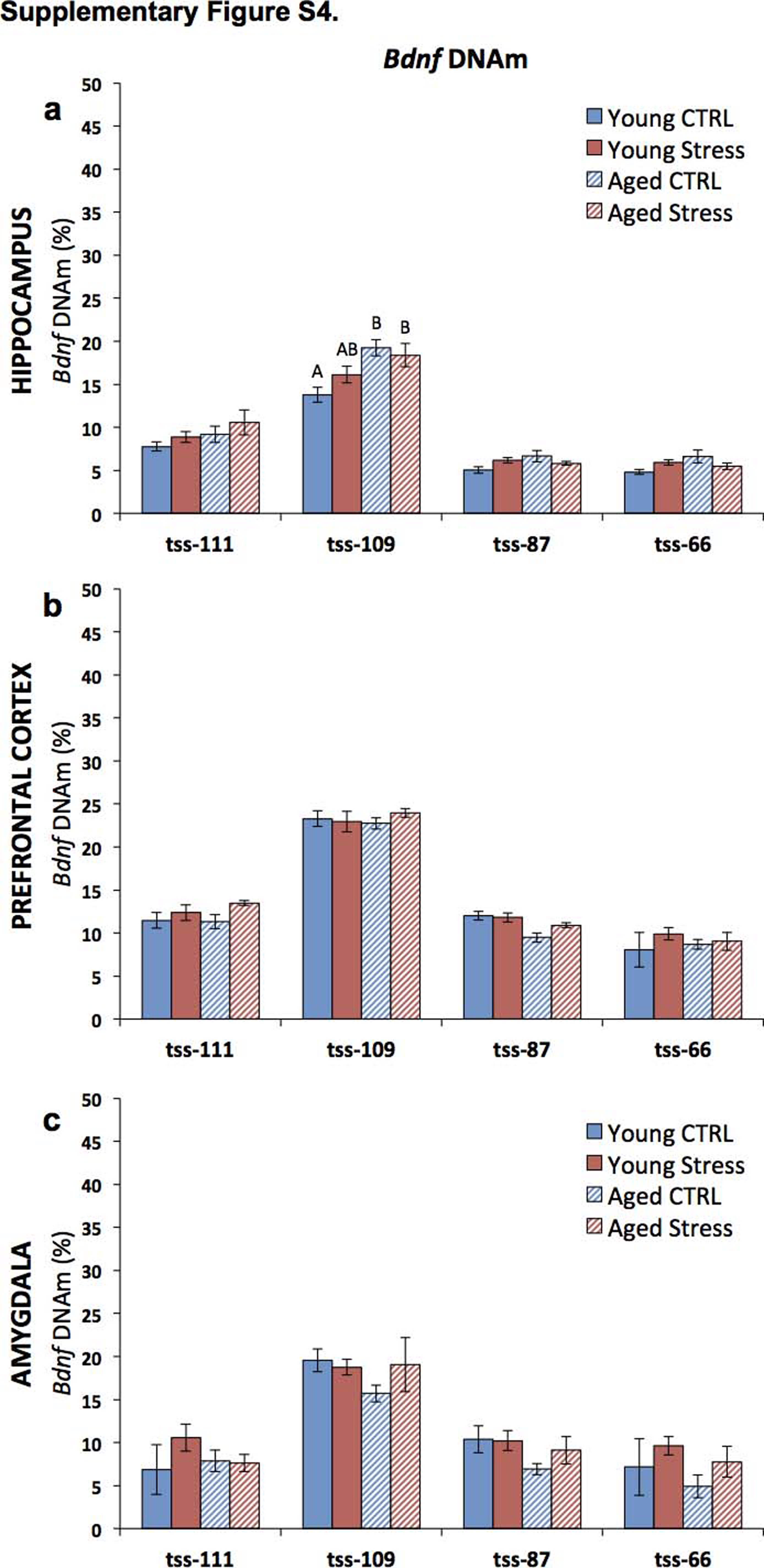

Supplement: Supplementary Figure 4 [file tp2016127x4.tif]

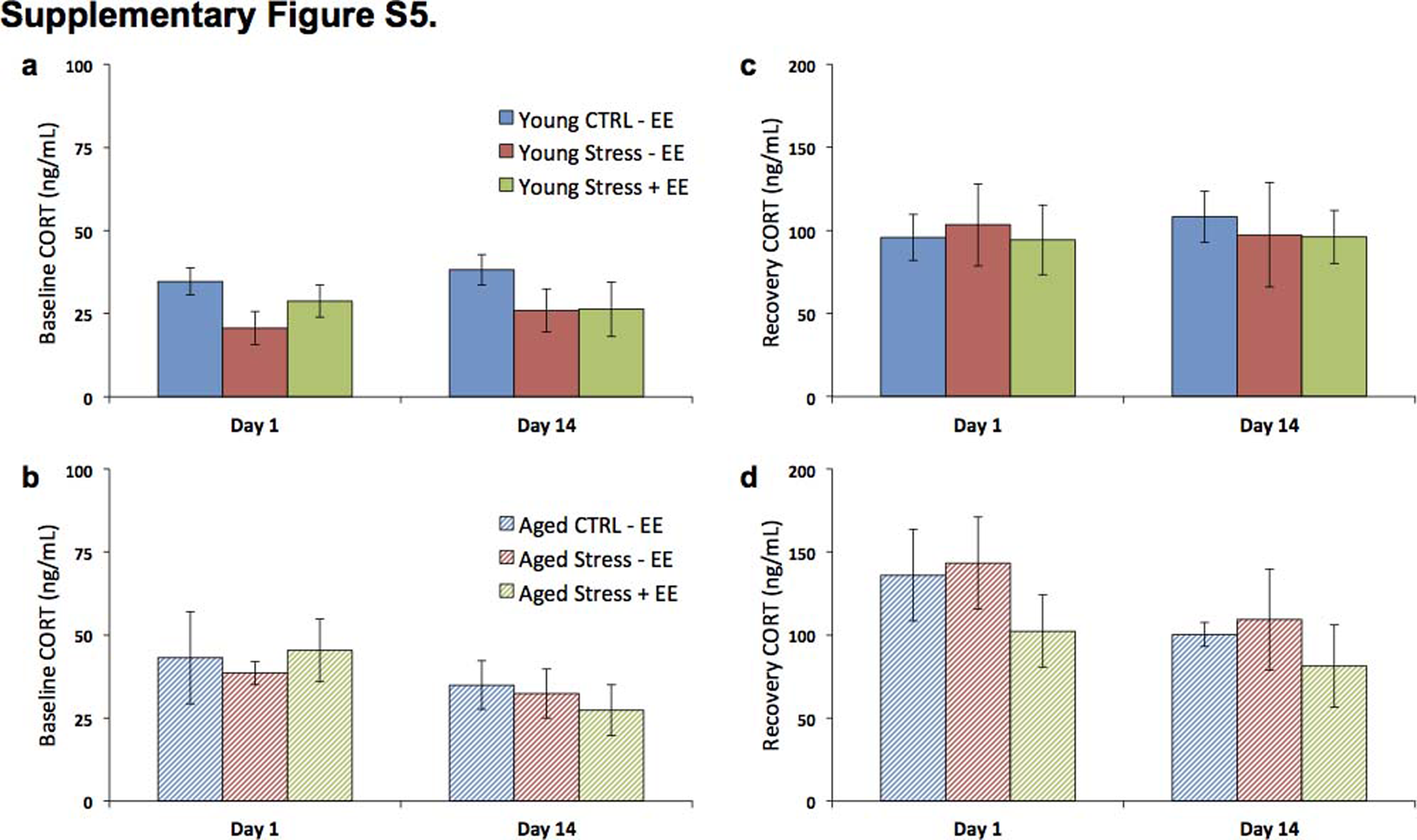

Supplement: Supplementary Figure 5 [file tp2016127x5.tif]

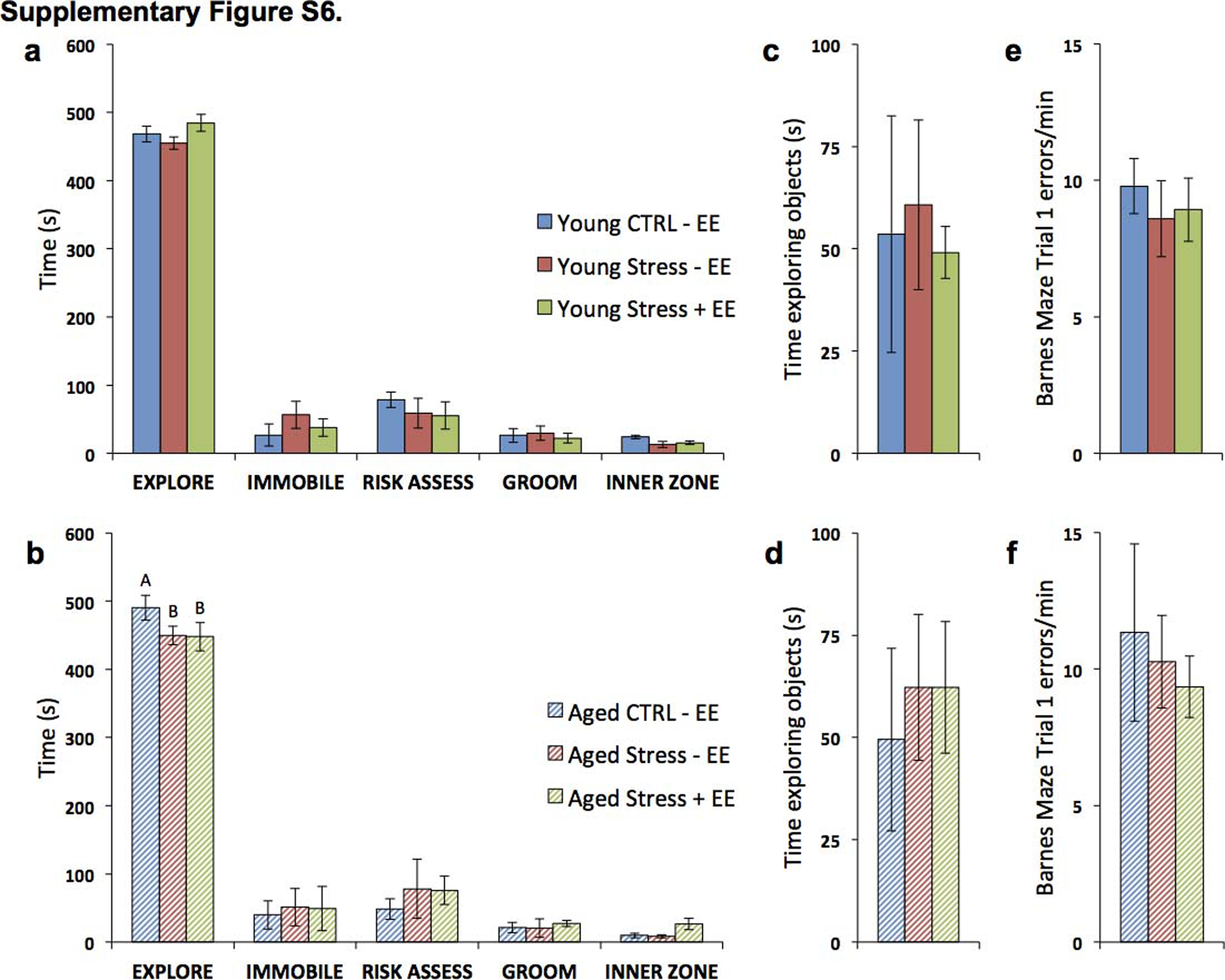

Supplement: Supplementary Figure 6 [file tp2016127x6.tif]
