# Supplementary material for: Effects of chronic variable stress on cognition and Bace1 expression among wild-type mice
Source: Transl Psychiatry. 2016 Jul 12;6(7):e854–. doi: 10.1038/tp.2016.127 (PMC5545713; doi:10.1038/tp.2016.127)
Supplement: Supplementary Figure Legends [file tp2016127x7.doc]

**Supplementary Figure S1.** (**a**) There were no effects of age or CVS on baseline CORT in restraint stress tests on day 1 and day 14. (**b**) There were no effects of age or CVS on recovery CORT in restraint stress tests on day 1 and day 14. Data represent mean ± SEM.

**Supplementary Figure S2.** Exploratory behaviors in the OF, NOR, and Barnes maze are unaffected by age or CVS. (**a**) There was no effect of age or CVS on behavior in the OF test. (**b**) In the acquisition trial of the NOR test, there were no effects of age or CVS on time spent exploring objects. (**c**) In the first trial of the Barnes maze, there were no effects of age or CVS on the number of errors made per minute. Data represent mean ± SEM.

**Supplementary Figure S3.** *Gsk3b* promoter region DNAm. (**a**-**c**) In the hippocampus, PFC, and amygdala, there were no effects of age or stress on methylation of the *Gsk3b* promoter region CpGs that were assessed. Data represent mean ± SEM.

**Supplementary Figure S4.** *Bdnf* exon 4 region DNAm. (**a**) In the hippocampus, aged mice had higher methylation at a CpG located at tss-109. (**b**,**c**) In the PFC and amygdala, there were no effects of age or stress on methylation of the CpGs that were assessed. Data represent mean ± SEM. For post hoc analysis, groups that do not share letters are significantly different (p<0.05).

**Supplementary Figure S5.** (**a**,**b**) Among young and aged mice, there were no group effects on baseline CORT in restraint stress tests on day 1 and day 14. (**c**,**d**) Among young and aged mice, there were no group effects on recovery CORT in restraint stress tests on day 1 and day 14. Data represent mean ± SEM.

**Supplementary Figure S6.** Exploratory behaviors in the OF, NOR, and Barnes maze are unaffected by age, CVS, or EE. (**a**) There were no group effects on the behavior of young adult mice in the OF. (**b**) Aged CTRL mice spent slightly longer exploring the OF compared to both Aged Stress and Aged Stress+EE mice. There were no other group effects among aged mice in the OF. (**c**,**d**) In the acquisition trial of the NOR test, there were no group effects on time spent exploring objects. (**e**,**f**) In the first trial of the Barnes maze, there were no group effects on the number of errors made per minute. Data represent mean ± SEM. For post hoc analysis, groups that do not share letters are significantly different (p<0.05).
